# From Data to Decisions: Leveraging Retrieval-Augmented Generation to Balance Citation Bias in Burn Management Literature

**DOI:** 10.3390/ebj6020028

**Published:** 2025-06-02

**Authors:** Ariana Genovese, Srinivasagam Prabha, Sahar Borna, Cesar A. Gomez-Cabello, Syed Ali Haider, Maissa Trabilsy, Cui Tao, Antonio Jorge Forte

**Affiliations:** 1 Division of Plastic Surgery, Mayo Clinic, Jacksonville, FL 32224, USAharprabha@gmail.com (S.P.); saharborna2021@gmail.com (S.B.); abrahamgomezcabello@gmail.com (C.A.G.-C.); dr.s.alihaider@gmail.com (S.A.H.); trabilsymaissa26@gmail.com (M.T.); 2 Department of Artificial Intelligence and Informatics, Mayo Clinic, Jacksonville, FL 32224, USA; tao.cui@mayo.edu; 3 Center for Digital Health, Mayo Clinic, Rochester, MN 55905, USA

**Keywords:** AI (artificial intelligence), large language model, RAG (retrieval-augmented generation), burn, plastic surgery, clinical decision support

## Abstract

(1) Burn injuries demand multidisciplinary, evidence-based care, yet the extensive literature complicates timely decision making. Retrieval-augmented generation (RAG) synthesizes research while addressing inaccuracies in pretrained models. However, citation bias in sourcing for RAG often prioritizes highly cited studies, overlooking less-cited but valuable research. This study examines RAG’s performance in burn management, comparing citation levels to enhance evidence synthesis, reduce selection bias, and guide decisions. (2) Two burn management datasets were assembled: 30 highly cited (mean: 303) and 30 less-cited (mean: 21). The Gemini-1.0-Pro-002 RAG model addressed 30 questions, ranging from foundational principles to advanced surgical approaches. Responses were evaluated for accuracy (5-point scale), readability (Flesch–Kincaid metrics), and response time with Wilcoxon rank sum tests (*p* < 0.05). (3) RAG achieved comparable accuracy (4.6 vs. 4.2, *p* = 0.49), readability (Flesch Reading Ease: 42.8 vs. 46.5, *p* = 0.26; Grade Level: 9.9 vs. 9.5, *p* = 0.29), and response time (2.8 vs. 2.5 s, *p* = 0.39) for the highly and less-cited datasets. (4) Less-cited research performed similarly to highly cited sources. This equivalence broadens clinicians’ access to novel, diverse insights without sacrificing quality. As plastic surgery evolves, RAG’s inclusive approach fosters innovation, improves patient care, and reduces cognitive burden by integrating underutilized studies. Embracing RAG could propel the field toward dynamic, forward-thinking care.

## 1. Introduction

### 1.1. Background

Burn injuries remain a critical clinical concern in the United States, accounting for approximately 29,165 hospital admissions annually, with 36.4% of these cases requiring surgical intervention [1]. These injuries present complex [2] and resource-intensive challenges [3] within the field of plastic surgery, often demanding a multidisciplinary approach that integrates critical care, physical rehabilitation, and psychological support [4]. The multifaceted nature of burn management requires a robust foundation of evidence-based knowledge [5]. However, the rapidly expanding and increasingly intricate body of medical literature creates substantial barriers [6] for plastic surgeons tasked with making timely, informed decisions. This growing volume of information [6], coupled with the demand for real-time clinical application [7], underscores the pressing need for innovative tools capable of synthesizing high-quality evidence with speed and precision. Such advancements hold the potential to enhance decision making, improve patient outcomes, and streamline the management of these complex cases.

Artificial intelligence (AI), defined as the ability of machines or computer systems to perform tasks traditionally requiring human intelligence [8], represents a transformative opportunity to address this challenge. Within plastic surgery, AI has already demonstrated its value in applications such as generating educational content [9], providing postoperative support for patients [10], and improving the accuracy of operative notes [11]. Among these tools, retrieval-augmented generation (RAG) is particularly promising. Large language models (LLMs) such as ChatGPT have the potential to reduce physician workload and support clinical decision making [12]. However, they are susceptible to inaccuracies or hallucinations, where they present misleading or incorrect information in a convincing manner, which can result in harm to patients [13]. By enhancing LLMs through the retrieval of targeted, high-quality information from external sources, RAG can optimize results by reducing inaccuracies and hallucinations [14], thereby offering a precise and efficient means of navigating the vast medical literature. This capability positions RAG as a valuable tool for supporting clinical decision making in high-stakes areas like burn management.

To fully unlock the potential of RAG, it is critical to evaluate how the quality of the source materials impacts its performance in synthesizing clinically relevant information. Citation metrics, commonly used as proxies for research quality, significantly influence which studies are deemed valuable [15] and trustworthy [16,17]. Additionally, citation counts play the largest role in Google Scholar’s ranking algorithm [18], showing the literature with more citations first. These metrics may guide clinicians toward highly cited papers [19] while potentially overlooking less-cited works that may provide valuable insights. Understanding the relationship between citation metrics and RAG’s performance is essential for reducing biases inherent in citation-based hierarchies, optimizing source selection, and ensuring the tool generates accurate and clinically applicable responses using texts that humans might deem less reliable. By moving beyond traditional reliance on citation counts, RAG introduces a more balanced and innovative approach to evidence synthesis.

Despite AI’s growing integration into plastic surgery, no studies have systematically investigated whether less-cited papers maintain the accuracy, readability, and response time of AI-generated outputs using RAG compared with highly cited works. Addressing this gap is essential to refining how AI tools synthesize medical knowledge and ensuring their alignment with the complex demands of clinical practice.

### 1.2. Research Objectives

To this end, our study evaluated RAG’s performance in answering clinical questions about burns and burn management, comparing the use of highly cited versus less-cited papers. Specifically, we investigated the following:

1. If citation metrics impact the accuracy of responses using RAG in burn management.
2. If the readability of RAG-generated responses is influenced by citation metrics.
3. If the use of highly cited papers affects RAG’s response time compared with less-cited sources.

By addressing these questions, we aim to provide critical insights into how citation metrics influence AI-driven evidence synthesis, with the goal of optimizing RAG’s performance, minimizing bias in source selection, and enhancing its application to streamline decision making and improve outcomes in the complex field of burn management.

## 2. Materials and Methods

### 2.1. Source Material Selection

Two groups of publications were identified to serve as source material for the LLM: 1 group consisted of 30 publications [2,20,21,22,23,24,25,26,27,28,29,30,31,32,33,34,35,36,37,38,39,40,41,42,43,44,45,46,47,48] with more than 100 citations (mean: 302.8), categorized as high-citation publications, and the other included 30 publications [49,50,51,52,53,54,55,56,57,58,59,60,61,62,63,64,65,66,67,68,69,70,71,72,73,74,75,76,77,78] with fewer than 50 citations (mean: 21.4), categorized as low-citation publications. Citation metrics were retrieved exclusively from Google Scholar in December 2024.

The thresholds of 100 and 50 citations were chosen to create a clear dichotomy between highly cited and less-cited works while ensuring that the selected studies were relevant and substantive within the field of burn management. Publications were selected from peer-reviewed sources, with non-peer-reviewed articles, editorials, and studies unrelated to burn management excluded. The dataset encompassed a broad spectrum of study designs, including systematic reviews, narrative reviews, retrospective and prospective cohort studies, case series, comparative studies, practice guidelines, expert consensus statements, and experimental research. This diversity of study types ensured a comprehensive dataset for evaluating AI-generated responses while reflecting the heterogeneity of the published literature in burn management.

To ensure content comparability and that both citation groups contained relevant literature that could address key areas of burn care, each high-citation publication was manually paired with a low-citation publication that covered a comparable clinical topic within burn management. By structuring the comparison in this way, we controlled for topic variability, helping to attribute observed differences in AI-generated responses to citation count rather than discrepancies in content availability. Once selected, each group of publications was compiled into a single PDF document, with all high-citation studies forming one PDF and all low-citation studies forming another. These PDFs were subsequently used as input for the RAG system.

This approach provided a structured, balanced comparison between citation groups while maintaining clinical relevance, allowing for a meaningful evaluation of citation bias in AI-driven evidence synthesis.

### 2.2. Question Development

A total of 30 clinical questions were developed to evaluate the performance of the LLM, spanning foundational knowledge, acute management, and advanced surgical techniques in burn care. To create a structured and unbiased evaluation framework, clinical questions were formulated to reflect key areas of burn assessment, resuscitation, wound healing, surgical techniques, and reconstructive strategies. These questions were intended to reflect the breadth of clinical challenges encountered in burn management, augmenting both relevance to real-world practice and rigor in evaluation. The questions ranged in complexity, from basic queries such as “What formulas can be used to calculate fluid resuscitation in burn patients?” to specialized surgical topics like “How are helical defects, resulting from burns to the helix of the ear, reconstructed surgically?”

Foundational questions targeted critical knowledge for acute care, such as fluid resuscitation and wound assessment, while more complex questions assessed the AI’s ability to retrieve and synthesize information required for nuanced surgical decision making and burn reconstruction. This diversity ensured coverage of common clinical scenarios as well as specialized topics such as the use of platelet-rich plasma or management of burn contractures. By spanning this range, the questions provided a robust framework to test the LLM’s ability to navigate the literature effectively, simulate the demands of clinical practice, and highlight the potential of RAG to support decision making in burn care.

After the question set was finalized, the selected high-citation and low-citation publications were reviewed to confirm that they contained sufficient information to address the questions. This verification step established that AI-generated responses could be meaningfully compared across citation groups without introducing topic bias. Any minor refinements to question wording were made only to enhance clarity and consistency, without altering the original scope or intent of the evaluation. All questions posed to the LLM can be found in Supplementary File S1.

### 2.3. Response Generation

**Figure 1:**
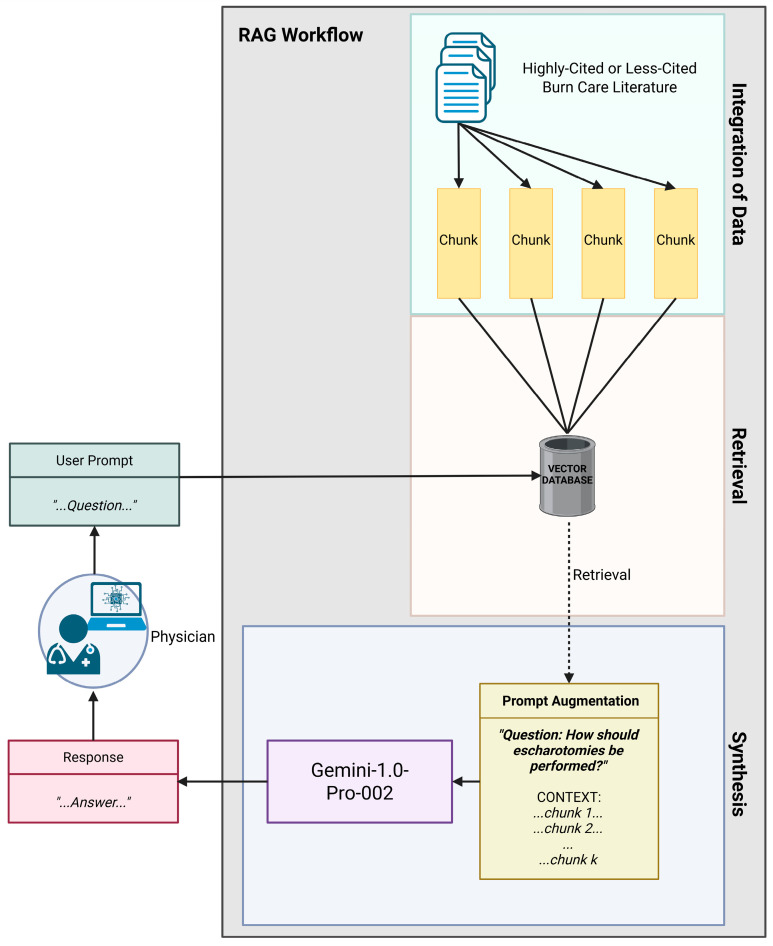
Leveraging retrieval-augmented generation (RAG)-based Gemini for burn care management assistance. Created with BioRender [80].

Questions were then posed to Gemini-1.0-Pro-002 on 11 December 2024, and responses were generated using RAG for each citation set. This model was chosen for its stability and predictability in its performance, suggested by its application in various domains [79]. Figure 1 demonstrates the study design utilizing the RAG workflow.

### 2.4. Accuracy Assessment

Accuracy was assessed using a 5-point scale, which provided a structured framework for evaluating the quality of the AI-generated responses: 1. The response was completely inaccurate or the model failed to answer the question; 2. The response was mostly inaccurate, containing significant errors or omissions; 3. The response was somewhat accurate, offering partial correctness or lacking critical details; 4. The response was mostly accurate, with minor errors or omissions that did not affect its overall validity; 5. The response was completely accurate, thoroughly addressing the question without any inaccuracies.

Two blinded independent reviewers, both full-time researchers in plastic surgery, used this scale to evaluate each response against the source text, providing an evidence-based assessment. This approach minimized subjectivity by directly comparing AI-generated content to established scientific findings. In cases where the reviewers assigned differing scores, a third reviewer analyzed the response and provided a final decision. This multi-reviewer approach minimized bias, ensured consistency, and upheld the rigor of the accuracy assessment process.

### 2.5. Readability Evaluation

Readability was assessed using two standardized metrics. The Flesch–Kincaid Grade Level was used to determine the minimum education level required to understand each response, with a 7th-grade reading level set as the benchmark for acceptability. This threshold aligns with the National Institutes of Health (NIH) recommendation that health materials be written at a 6th- to 7th-grade level to ensure accessibility [81]. Although healthcare professionals typically read at a higher level [82], we selected this benchmark to account for the fast-paced clinical environment, where clear and easily digestible information is essential for efficient decision making.

The Flesch Reading Ease Score, ranging from 0 to 100, assessed the overall readability, with higher scores indicating easier-to-read text. These metrics were calculated using a freely available online tool [83], ensuring objectivity and consistency across all evaluations.

### 2.6. Response Time Assessment

Response time, defined as the duration in seconds required for the LLM to generate each response, was recorded for every query. This parameter provided insight into the efficiency of the model when using high-citation versus low-citation source materials.

### 2.7. Statistical Analysis

All performance variables were reported as median (range) and mean (standard deviation). Wilcoxon rank sum test was used to compare these performance measurements scored for questions from high− vs. low-cited papers. All tests were two-sided, with *p* value < 0.05 considered statistically significant. The analysis was performed using R4.2.2.

A Fleiss Multi-Rater Kappa Intraclass Correlation Coefficient (ICC) analysis was performed using a Two-Way Mixed model with absolute agreement to determine the level of agreement between the two reviewers. This was performed for all responses from both high-citation and low-citation datasets. A 95% confidence interval was used.

## 3. Results

### 3.1. Accuracy Results

When prompted with questions about burn management, Gemini-1.0-Pro-002 generally produced accurate responses using RAG for both high- and low-citation datasets. The mean accuracy score for the high-citation set was 4.6 (SD = 0.7), compared with 4.2 (SD = 1.4) for the low-citation set. The ICC value for the combined dataset (n = 60) was 0.601 (95% CI: 0.435–0.767), indicating moderate agreement between the reviewers.

Both citation datasets performed well, achieving a score of 5 for many questions related to simple retrieval, management strategies, and complex clinical reasoning (e.g., “How are helical defects, resulting from burns to the helix of the ear, reconstructed surgically?”). However, both datasets also had instances of lower performance.

Scores of 3 or less occurred in both groups, including three cases in the low-citation set where the model was unable to generate a response despite the relevant information being present in the dataset (10%). For example, when asked about management of extensive total body surface area (TBSA) full-thickness burns, Gemini responded: “[the source text] does not mention full-thickness burns or have information on how to treat a burn with an extensive total body surface area (TBSA)”. However, the provided text stated: “In patients with extensive %TBSA, current standard of care is to use serial meshed grafting, or when it is available and affordable, cultured epithelial autografts” [62].

Additionally, some responses that did not fail completely still received lower scores due to incomplete retrieval of information. For instance, in response to the question “When should burn scars be treated with lasers to achieve the best results?”, the high-citation dataset generated “early intervention through laser treatment leads to better outcomes for burn scars”. However, it failed to retrieve a critical timeframe specified in the source text: “within weeks and months of injury” [44].

All responses that received a score of 1 or 2 either failed to generate a substantive answer or provided vague, overly cautious, or incomplete statements, often deflecting the question or omitting key clinical recommendations that were present in the source material. For example, in response to the question, “What is the treatment of choice for burn wound contracture release?”, the low-citation dataset produced a non-committal response that emphasized general surgical techniques and deferred to clinician judgment, but did not extract the text’s recommendation favoring full-thickness skin grafts [56]. These outputs did not meet the definition of hallucination, as they did not contain fabricated or false information, but instead reflected an underutilization of available content.

**Figure 2:**
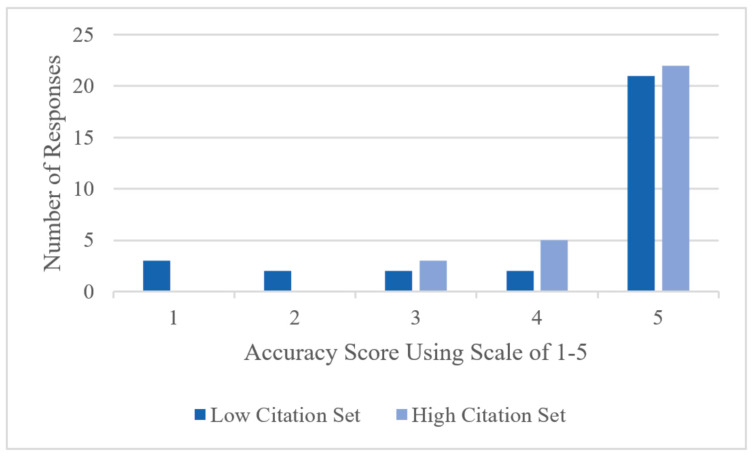
Accuracy of low- and high-citation sources for retrieval-augmented generation using scale of 1–5. Created with Microsoft Excel.

Despite these retrieval challenges, 90% were rated as mostly or completely accurate (scores of 4 or 5) among the high-citation responses, compared with 76% in the low-citation dataset. The difference in accuracy between the low− and high-citation datasets was not statistically significant (*p* = 0.49). The distribution of scores is presented in Figure 2.

### 3.2. Readability Results

The readability of the questions was assessed using the Flesch–Kincaid Grade Level and Flesch Reading Ease formulas. For the low-citation dataset, the mean Flesch–Kincaid Grade Level was 9.5 (SD = 2.7), with a range from 5.0 to 15.4. Similarly, the high-citation dataset demonstrated a mean Flesch–Kincaid Grade Level of 9.9 (SD = 2.4), ranging from 5.0 to 14.8. Both datasets exceeded the 7th-grade reading level benchmark, indicating a generally higher level of complexity, and no statistically significant difference was found between the two groups (*p* = 0.29).

Using the Flesch Reading Ease formula, the low-citation dataset had a mean score of 46.5 (SD = 18.8), with scores ranging from 7.5 to 72.5. The high-citation dataset had a slightly lower mean score of 42.8 (SD = 16.2), with scores ranging from 3.8 to 72.3, with no statistically significant difference (*p* = 0.26). These results suggest that both datasets contained questions with moderate to low readability.

### 3.3. Response Time Results

The response times for the questions were analyzed to compare the high-citation and low-citation datasets. The high-citation dataset had a mean response time of 2.8 s (SD = 1.4), with a range from 0.9 to 5.6 s. The low-citation dataset demonstrated a slightly lower mean response time of 2.5 s (SD = 1.3), with a range from 0.9 to 4.7 s. The difference in mean response times between the two datasets was not statistically significant (*p* = 0.39). Table 1 demonstrates the results for accuracy, readability, and response time.

## 4. Discussion

### 4.1. Summary of Key Findings

This study marks a substantial advancement in how we approach source selection for retrieval-augmented generation systems, with direct relevance to burn management and a potential ripple effect across broader clinical decision-making domains. Our analysis reveals that RAG can produce accurate, readable, and efficient responses to clinical burn-related inquiries using both highly cited and less-cited research. While highly cited sources displayed a modest edge in consistency, no statistically significant differences emerged across accuracy, readability, or response time. These results challenge the entrenched reliance on citation-rich literature in evidence synthesis, demonstrating that thoughtfully curated, less-cited materials can yield performance comparable to their more frequently referenced counterparts.

### 4.2. Interpretation of Results

The slightly higher mean accuracy among highly cited sources (4.6 vs. 4.2) may initially suggest a qualitative advantage. However, the lack of statistical significance (*p* = 0.49) indicates that this variance could stem from sample characteristics rather than any intrinsic superiority of heavily cited studies. Furthermore, Lindgren observed no significant difference in research design or methodological approaches when comparing highly and less-cited papers [84], indicating factors beyond methodological variability, such as text structure, location, or information density, may have had a greater impact on the mean accuracy scores.

Some responses received lower accuracy scores not because the answers were absent from the literature, but likely due to semantic inconsistencies between the prompt and source text or a lack of contextual understanding. In a few cases, Gemini was unable to generate a response despite the relevant details being explicitly present in the dataset, suggesting challenges in how the model retrieved and prioritized information. Additionally, some responses that were generated lacked specificity, failing to extract the key details necessary for a fully accurate answer. These outcomes likely reflect not only the interplay between source text characteristics and prompt formulation, but also broader assimilation challenges in retrieval-augmented generation.

RAG systems depend on document chunking and relevance ranking algorithms that may inadvertently prioritize less-informative segments or omit essential content, particularly when retrieval granularity is coarse or semantically misaligned with the query [85]. Even when relevant information is retrieved, synthesis may be constrained by the model’s fixed context window, which restricts access to the broader information landscape [86]. These technical limitations may have contributed to inconsistencies in response specificity and completeness across the citation groups, even when overall accuracy scores were statistically similar. These assimilation dynamics help explain the variability in response specificity across citation groups.

Beyond these assimilation-related challenges, additional factors unrelated to retrieval may have influenced response quality. Accuracy variations across citation groups in this study also highlight the influence of language model pretraining, including prior exposures and biases in how certain studies are discussed within the broader literature. While retrieval-augmented generation systems are designed to ground outputs in source material, the underlying training data of large language models still shape response formulation [85]. These models are trained on vast and heterogeneous datasets, which include both high-quality scholarship and unreliable or outdated information [87]. As a result, even when RAG retrieves relevant studies, the model’s pretraining biases may influence which details it prioritizes or omits, potentially amplifying widely cited perspectives while underrepresenting emerging or niche findings. This phenomenon is particularly relevant in source selection, as curating high-quality literature does not fully eliminate biases if the language model inherently favors certain interpretations over others [88]. To mitigate these risks, the quality of source texts should be critically assessed prior to selection, with particular attention to potential biases in the study design, reporting, and framing of clinical evidence. Addressing these challenges requires not only refining retrieval mechanisms but also a deeper understanding of how model pretraining, retrieval ranking, and synthesis strategies interact to shape AI-generated evidence.

While citation count did not significantly impact the accuracy, readability, or response time of RAG-generated outputs in this study, highly cited papers are more widely disseminated across academic and public sources, making them more likely to appear in the pretraining data of large language models. Moreover, Algaba et al. found that LLMs exhibit a strong bias toward highly-cited sources even after controlling for other variables such as the year of publication, author count, and title length [89]. Although RAG reduces direct reliance on pretraining biases by grounding responses in the selected literature, citation frequency could still influence retrieval ranking, synthesis patterns, or response framing by the LLM, particularly if frequently referenced sources are overrepresented in the training corpus or cited disproportionately within the retrieved documents. These recursive citation patterns—akin to a form of field-level self-citation—may subtly shape the model’s attention toward dominant viewpoints, potentially biasing both the results of this study and future model behavior. This concern is especially relevant when generating plain language summaries, where the amplification of prevailing narratives may overshadow less-cited but clinically valuable insights. Although citation-related dynamics may still shape how information is prioritized, the absence of significant performance differences between the citation groups in this study suggests that RAG-based retrieval can help mitigate disparities, supporting the feasibility of AI-driven evidence synthesis grounded in a broader, citation-diverse knowledge base.

This insight is critical: less-cited studies, when properly vetted for relevance, can contribute significantly to high-quality AI outputs. This contrasts with popular opinion, emphasized by findings such as those by Teplitskiy et al., which highlighted that lower-cited papers are often perceived as having reduced quality and may elicit less-meaningful engagement [19]. By reframing the conversation around source selection and increasing awareness of these inherent biases, our results advocate for a more inclusive and balanced approach to evidence curation. Rather than perpetuating a reliance on citation volume as a marker of value, this study highlights the potential of integrating emerging and underutilized research to enrich AI-driven evidence synthesis.

Notably, readability remained consistent across both groups, with average Flesch–Kincaid levels of 9.5 for the low-citation group and 9.9 for the high-citation group, indicating that citation metrics did not influence linguistic complexity in AI-generated outputs. Interestingly, prior research has found that more complex and less-readable texts are more likely to receive citations [90], and abstracts of highly cited works often feature more professional and intricate language [91]. This suggests that while citation-heavy studies may exhibit greater linguistic complexity, the RAG system’s ability to synthesize information helps to neutralize such differences, resulting in outputs with similar readability regardless of source citation status.

Although both sets of responses surpassed the recommended 7th-grade reading level, this reflects a style more aligned with professional discourse than the swift, digestible summaries often preferred in clinical settings. While large language models are adaptable and capable of adjusting readability when prompted [92], prior research has shown that they often struggle to consistently tailor responses to specific reading levels, even with explicit instructions [92,93]. In this study, no specific readability constraints were applied, meaning that response complexity was dictated solely by how the model synthesized the retrieved content. The observed readability levels suggest that, in the absence of targeted prompts, the model defaults to a professional tone rather than an easily digestible summary. With improvements, this adaptability could be leveraged in clinical applications, where tailoring responses to different audiences may enhance the accessibility and utility of AI-generated medical information.

Readability is not merely a hallmark of patient education; in time-sensitive clinical environments, delivering concise, easily comprehensible information can streamline decision making and reduce cognitive burden on clinicians. Studies have shown that clinicians prefer synthesized evidence over original research [94] and may struggle to accurately interpret complex data displays [95]. To be effective, clinical tools must provide information that is easy to access, straightforward to navigate, and fast to implement [96]. The RAG system’s ability to synthesize information into similarly readable formats, regardless of source citation status, positions it as a valuable tool for addressing these critical needs.

Lastly, the similar response times (2.8 s for high-citation vs. 2.5 s for low-citation sources) confirm that expanding the pool of evidence need not compromise efficiency. Notably, the high-citation dataset consisted of 313 pages, while the low-citation dataset contained 278 pages, yet this difference in document size did not lead to a statistically significant difference in response time. This suggests that RAG processing time remained stable despite variations in input length, reinforcing its feasibility for real-time clinical applications. By validating the feasibility of incorporating less-cited materials without performance trade-offs, the study underscores the potential for AI-driven tools to move beyond traditional, citation-based hierarchies. In doing so, it fosters the development of more adaptable, equitable, and ultimately more impactful approaches to evidence synthesis in clinical medicine.

### 4.3. Strengths and Limitations

This study presents several notable strengths that enhance the validity and rigor of its findings. A key strength lies in the systematic approach to source selection and evaluation, which ensured a balanced comparison between highly cited and less-cited materials. By using blinded assessments for accuracy and standardized readability metrics, the study minimized bias and provided objective, reproducible measures of performance. Additionally, the study’s focus on burn management, a complex and resource-intensive area of plastic surgery, emphasizes its clinical relevance, particularly in demonstrating the potential of retrieval-augmented generation to address real-world challenges in evidence synthesis.

However, the study also has important limitations that should be acknowledged. First, the analysis was limited to a single clinical domain (burn management) and employed only one large language model, Gemini-1.0-Pro-002. While this focused approach allowed for a controlled investigation, the results may not generalize to other AI models or specialties within plastic surgery. Future research is necessary to replicate these findings across diverse medical contexts and with alternative AI systems to confirm the broader applicability of RAG tools.

Another key limitation relates to the sample size and resulting statistical power of this study. Although no statistically significant differences were observed in accuracy, readability, or response time between high− and low-citation sources, the relatively small number of questions (n = 30) may not provide sufficient power to detect subtle or moderate effects. Additionally, qualitative differences of responses may exist that were not captured by our scoring rubric. With a larger dataset, it is possible that differences in RAG performance metrics could emerge as statistically meaningful. Until such data are available, our findings should be interpreted as preliminary.

Furthermore, the thresholds for “highly cited” and “less-cited” sources were chosen somewhat arbitrarily to create a clear dichotomy for analysis. While this decision enabled a straightforward comparison, it does not account for the continuous nature of citation metrics or potential nuances within the spectrum of study quality. Expanding future studies to include a wider range of citation thresholds and additional quality metrics, such as methodological rigor or level-of-evidence hierarchies, would provide a more comprehensive understanding of how source characteristics influence AI performance.

Additionally, while low-scoring responses were reviewed qualitatively, the true hallucination rate cannot be determined based on the accuracy rubric used in this study.

Next, while all prompting was conducted on a single day to minimize variability, response time measurements may still have been influenced by external factors such as server load, network congestion, or bandwidth fluctuations. These variables were not explicitly controlled and could affect generalizability.

Finally, the study prioritized isolating the impact of citation-based selection over exploring other factors that could affect RAG outputs. While this focus addresses a critical gap, it also highlights the need for subsequent investigations to incorporate broader quality indicators and consider the interplay between citation metrics and other attributes. Another potential limitation lies in the process of prompt selection. While every effort was made to design clear and specific queries, the possibility remains that ambiguous or imprecise prompts could influence RAG outputs, potentially resulting in incomplete or less-relevant responses. This highlights the need for ongoing refinement of input designs to ensure outputs are consistently accurate and clinically meaningful.

Despite these limitations, this research serves as a valuable proof of concept, demonstrating the feasibility of using less-cited materials in AI-driven evidence synthesis and providing a strong foundation for future advancements in clinical decision support tools.

### 4.4. Implications and Future Directions

The implications of these findings extend well beyond the specific context of burn management, suggesting that clinicians and researchers need not remain bound by citation-based hierarchies when leveraging RAG systems. By demonstrating that less-cited sources can perform comparably to highly cited counterparts, this study highlights the potential for a broader, more equitable approach to evidence integration. Incorporating a diverse range of literature—some of which may include innovative techniques, emerging evidence, or niche insights—can enrich evidence synthesis without sacrificing accuracy, efficiency, or readability. This inclusivity could directly inform best practices for curating AI training datasets, ensuring that relevant studies are integrated regardless of their citation volume. Such an approach could support physicians in other areas of plastic surgery, such as craniofacial reconstruction, complex wound healing, and microsurgical interventions, where rapid access to nuanced information is essential for effective treatment planning.

While broadening the scope of training datasets is essential, the criteria used to select the source material must also be carefully considered. Journal impact factors, though often used as a proxy for publication quality, reflect aggregate citation behavior and may favor disciplines with higher publication volumes or broader audiences rather than methodological rigor or clinical relevance. Over-reliance on such metrics could unintentionally reinforce established hierarchies and sideline emerging or underrepresented research. Future LLM training pipelines should move beyond journal-level metrics and instead prioritize source-level indicators such as study design quality, peer-review transparency, and clinical applicability to support more balanced, trustworthy evidence synthesis.

To build on this work, future efforts should focus on developing refined source selection strategies that incorporate additional quality indicators such as methodological rigor and risk of bias. This would improve the adaptability of RAG systems, allowing them to dynamically respond to evolving literature landscapes and integrate emerging research that has not yet gained significant citations. Additionally, exploring domain-specific tailoring and enhancing natural language processing techniques will be critical for improving clinicians’ trust in and adoption of these tools. User-centered interface improvements designed to align with the workflows of different healthcare professionals could further enhance their effectiveness and accessibility.

Moreover, future studies should address the diverse needs of RAG’s end users, with accuracy as a central priority. For clinicians, advanced RAG methods are required to prevent errors such as hallucinations or omissions, with evaluations focusing on diagnostic concordance, decision-making efficiency, and seamless integration into electronic health records. For patient-facing applications, accuracy must be paired with readability to ensure the information is accessible and actionable, especially in settings with varying levels of health literacy.

To address recursive citation patterns, diversity-enforcing techniques, including constraints in retrieval algorithms and novelty detection, should be utilized. During training, models should be exposed to a balanced distribution of citations using augmented datasets that emphasize diversity. Transparency in outputs, where citation patterns are visible to users, will enable the informed evaluation of evidence. Additionally, framing queries to target specific sections of papers—such as Background, Methodology, Results, and Discussion—and using structured queries for cross-verification will aid comprehensive and unbiased information extraction.

To ensure adaptability across clinical contexts, a consistent, domain-specific framework for evaluating RAG systems should be developed. This framework should include standardized benchmarks for accuracy, usability, and readability alongside iterative feedback from both clinicians and patients. By grounding RAG development in such a structured and inclusive approach, these systems can evolve to deliver equitable, high-quality evidence tailored to the complex and varied needs of healthcare professionals and patients alike.

With continued refinements, RAG has the potential to support clinical decision making, provide patient education, and streamline information synthesis in time-sensitive settings. The development of these tools lays the groundwork for improving patient care and outcomes in the future by supporting physicians with more diverse, relevant, and accessible information resources. By enabling physicians to select sources independent of citation bias, RAG systems can empower clinicians with a broader, more diverse evidence base that aligns with real-world practice needs.

## 5. Conclusions

This study demonstrates the potential of retrieval-augmented generation systems to streamline data retrieval in healthcare, producing accurate, readable, and efficient responses to clinical questions in burn management regardless of source citation status. By showing that less-cited materials can perform on par with highly cited ones, the findings challenge traditional citation hierarchies and support a more inclusive approach to evidence synthesis. This capability addresses the growing demand for timely, reliable information in complex fields like plastic surgery, offering a practical tool to reduce the cognitive burden on clinicians and support informed decision making.

## Figures and Tables

**Table 1:** Summary of results comparing accuracy, readability, and response time for RAG using high- and low-citation sources.

	High-Citation Set (N = 30)	Low-Citation Set (N = 30)	*p* Value
Accuracy			0.49
Mean (SD)	4.6 (0.7)	4.2 (1.4)	
Median (range)	5.0 (3.0, 5.0)	5.0 (1.0, 5.0)	
Response Time (seconds)			0.39
Mean (SD)	2.8 (1.4)	2.5 (1.3)	
Median (range)	2.5 (0.9, 5.6)	1.9 (0.9, 4.7)	
Flesch–Kincaid Grade Level			0.29
Mean (SD)	9.9 (2.4)	9.5 (2.7)	
Median (range)	10.4 (5.0, 14.8)	9.1 (5.0, 15.4)	
Flesch Reading Ease			0.26
Mean (SD)	42.8 (16.2)	46.5 (18.8)	
Median (range)	41.7 (3.8, 72.3)	50.4 (7.5, 72.5)	

## Data Availability

Data are contained within the article or Supplementary Materials.

## Supplementary Materials

The following supporting information can be downloaded at: https://www.mdpi.com/article/10.3390/ebj6020028/s1, Supplementary File S1. High Citation LLM Response Using RAG; Supplementary File S1 contains the 30 clinical questions used in this study, along with the RAG-generated responses sourced from low- and high-citation datasets, presented side by side for direct comparison.

## Abbreviations

The following abbreviations are used in this manuscript:

RAG	Retrieval-augmented generation
LLM	Large language model
